# Hypothesis of the Causal Mechanisms Between Gut Microbiota and Neurodegenerative Diseases: An Elucidation from Evolutionary Perspective and Metabolic Consideration

**DOI:** 10.3390/metabo16050337

**Published:** 2026-05-18

**Authors:** Guangyan Tang, Liwen Guo, Zhiwei Liu, Yuan Quan

**Affiliations:** Hubei Key Laboratory of Agricultural Bioinformatics, College of Informatics, Huazhong Agricultural University, Wuhan 430070, China; gytang@mail.hzau.edu.cn (G.T.); 18536953310@163.com (L.G.); 2025317120088@webmail.hzau.edu.cn (Z.L.)

**Keywords:** gut microbiota, neurodegenerative diseases, human accelerated regions, metabolites, causality

## Abstract

Growing evidence links gut microbiota dysbiosis to neurodegenerative diseases (NDs) such as Alzheimer’s disease and Parkinson’s disease, yet the field remains dominated by correlational observations rather than experimentally validated causal mechanisms. In this hypothesis-generating Perspective, we propose that causal inference in microbiota-associated neurodegeneration may be strengthened by combining two complementary lenses: evolutionary biomedicine and microbial metabolism. Because evolutionary information carries intrinsic temporal and causal structure, it can provide biological prior knowledge for inferring causal mechanisms of diseases. Human Accelerated Regions (HARs), genomic loci conserved across mammals but rapidly divergent in the human lineage, offer an anchor for identifying human-specific host–microbe co-evolutionary units relevant to NDs. We further hypothesize that microbial metabolites represent one class of mechanistically testable intermediates linking host genetic background, gut microbial ecology, and neurodegenerative phenotypes. This integrated evolutionary-metabolic perspective offers a tractable path from correlation toward mechanism in gut microbiota–ND research.

## 1. Introduction

During the millions of years of co-evolution between humans and microbes, both parties have gradually formed a dynamic equilibrium and symbiotic system through complex bidirectional regulatory mechanisms [1]. As the host, humans shape microbial niches by providing a constant-temperature environment, nutrient substrates, and biological barriers. In turn, microbes play a crucial role in maintaining host homeostasis and the development of diseases. The gut harbors the largest and most complex microbial ecosystem in the human body. To date, approximately 3500 species of microbes have been identified in the human gut, forming a vast and sophisticated “second genome” [2]. Gut microbes communicate with the central nervous system through the microbiota–gut–brain axis and play an important role in brain development and cognitive regulation [3].

Although microbiota alterations have been repeatedly reported in Alzheimer’s Disease (AD), Parkinson’s Disease (PD), and Amyotrophic Lateral Sclerosis (ALS) [4], as well as related neurodegenerative diseases (NDs) [3,5,6,7,8], most research in this field remains at the descriptive or correlational analysis stage, which only demonstrates a statistically significant correlation between gut microbiota and NDs; the causal relationship between the two remains highly controversial [9,10,11,12,13]. If certain host–microbiota interactions are truly causal, they may yield actionable biomarkers and therapeutic targets; if they are instead secondary consequences of disease progression, then microbiota-based interventions may produce limited or inconsistent benefit.

**Figure 1:**
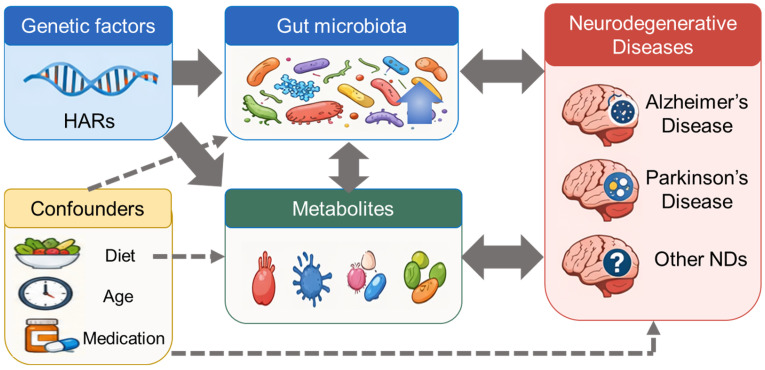
Schematic overview of causal mechanisms between HARs, gut microbiota, microbial metabolites, and neurodegenerative diseases. Human accelerated regions (HARs), as representative host genetic factors, can influence the composition and function of the gut microbiota. Altered microbial communities subsequently reshape the metabolic output of the gut ecosystem, including lipopolysaccharides (LPS), short-chain fatty acids (SCFAs), tryptophan-derived metabolites, and bile acids. These metabolite-mediated host–microbiota interactions may contribute to the development and progression of neurodegenerative diseases, including Alzheimer’s disease (AD), Parkinson’s disease (PD), and other neurodegenerative disorders (NDs).

In this Perspective, we propose that the possible causality in microbiota-associated neurodegeneration can be evaluated across host evolutionary genetics, microbial metabolite output, and disease phenotype (Figure 1). Within this framework, human accelerated regions (HARs) may provide host-side evolutionary anchors for prioritizing susceptibility genes, whereas microbial metabolites act as mechanistically testable effectors. We therefore propose that an evolutionary and metabolite-centered framework is better suited than conventional association-based models for identifying pathogenic mediators and therapeutically relevant targets in NDs. However, the HAR–microbiota–metabolite–ND causality proposed here interpreted as a hypothesis-generating framework. Some components, such as microbiota–ND associations and selected metabolite-mediated inflammatory mechanisms, are supported by clinical and experimental literature, whereas other components, particularly specific HAR–microbiota links, remain indirect and require independent validation. The model is modified by major confounders and contextual factors, including age, diet, medication, lifestyle, geography, disease stage, sex, host immune status, and sampling/measurement platforms [14,15,16].

## 2. Challenges in Deciphering Causal Mechanisms

Despite the growing literature on microbiota–ND associations, whether gut dysbiosis is a driver, a modifier, or merely a consequence of neurodegeneration remains unresolved [9,10,11,12,13]. On one hand, some studies suggest that gut dysbiosis may be a prodromal event of NDs, where the microbiota induces pathological cascades by regulating the immune system, neurotransmitter levels, and metabolic states. For instance, an increase in pro-inflammatory microbiota can disrupt the intestinal barrier and trigger systemic inflammation, creating conditions for neurodegeneration. On the other hand, some argue that changes in gut microbiota are merely a concomitant phenomenon or a host adaptive response during disease progression, rather than a direct pathogenic factor. To date, only a few cases have confirmed that gut microbes can directly cause disease, such as *Helicobacter pylori* inducing gastric ulcers and gastric cancer [17], and *Clostridioides difficile* mediating infectious diarrhea [18]. Furthermore, highly context-dependent confounding factors, ranging from host genetics and age to diet and medication exposure, can profoundly influence gut microbiota composition, significantly complicating efforts to decipher the underlying pathogenic mechanisms [14,15,16,19,20,21]. Given the multifaceted nature of the gut–brain axis, which involves immune modulation, neurotransmitter metabolism, and oxidative stress; relying solely on traditional correlation studies makes it difficult to systematically analyze the mechanisms of their association.

In biomedical research, identifying causal associations is crucial for developing microbiota-targeted intervention strategies [10,11,12,22]. If certain microbes are proven to have a causal role, their regulation would become an effective therapeutic approach; conversely, if they are only concomitant phenomena, corresponding interventions are unlikely to yield the expected efficacy and may even lead to side effects. For example, a recent double-blind randomized controlled trial (RCT) in Finland showed that while a single anaerobically prepared Fecal Microbiota Transplantation (FMT) was safe for PD patients, it failed to provide clinical improvement in core motor symptoms, and the FMT group experienced more gastrointestinal adverse events [23]. Another placebo-controlled phase 2 trial also evaluated FMT in mild-to-moderate PD, further highlighting both the translational promise and the current inconsistency of microbiota-based intervention outcomes [24]. The current failures to establish a rigorous causal chain in the study of gut microbiota and NDs has, to some extent, hindered the clinical translation of gut microbiota-based intervention strategies.

Traditional observational studies (such as cohort studies) can reveal epidemiological associations between gut microbiota and diseases. However, they are susceptible to confounding factors like diet, drugs, and lifestyle, and it is difficult to exclude reverse causality. To address these limitations, future research must shift toward a standardized and rigorously adjusted framework. This approach should prioritize the collection of comprehensive, standardized clinical metadata (e.g., sample handling, omics platforms, and population structure) and incorporate robust causal adjustment designs, such as directed acyclic graph (DAG)-guided modeling, sensitivity analyses, negative-control designs, and cross-cohort replications, to systematically reduce confounding bias and improve the interpretability of host–microbiota interactions. Currently, several experimental methods are available for studying the causality between gut microbiota and diseases. FMT, which involves transplanting fecal microbiota from healthy donors into recipients to observe the impact on host phenotypes, is one of the important experimental strategies for directly verifying the causal effects of gut microbes [11]. However, FMT still faces controversies regarding ethics, safety, and individual response variations, which limits its application. Another common method is the Human-Microbiota-Associated (HMA) rodent model, which assesses the impact of donor microbiota on the pathological phenotype of recipient animals by transplanting human donor gut microbiota into germ-free mice or other rodents. Although HMA models have confirmed causal relationships between specific microbes and diseases in some studies, they possess certain physiological and ecological limitations [12]: some human microbes struggle to colonize stably in mice, affecting experimental reproducibility; even if colonization is successful, the microbial community structure and metabolic environment of the recipient animal still differ significantly from those of humans, limiting the generalizability of the results. Therefore, experimental causal inference methods still have significant limitations.

In past decades, a series of statistical computational methods have been proposed for causal inference between the gut microbiota and diseases, with representative methods including Mendelian Randomization (MR) and Mediation Analysis [10]. As a causal inference method based on genetic instrumental variables (IVs), MR effectively circumvents confounding bias and reverse causality in traditional epidemiological studies by using genetic variations significantly associated with microbial features (e.g., microbial abundance) as instrumental variables. However, MR relies on strict genetic IV assumptions. The effect sizes of genetic variants obtained from studies such as mGWAS (microbial Genome-Wide Association Study) are generally low (*p* < 10^−5^) and exhibit widespread linkage disequilibrium (r^2^ > 0.8), making it difficult to satisfy the specificity requirements of instrumental variables. Mediation analysis is a statistical method used to study whether the causal effect of an exposure on an outcome is mediated by an intermediate variable. It can use linear structural equation modeling to process high-dimensional metagenomic data to elucidate the causal role of microbes in disease occurrence [25]. However, traditional mediation analysis assumes linear relationships and continuous variables, whereas ND outcomes are often non-continuous, requiring a non-parametric framework for causal mediation analysis, which has high computational complexity and low statistical power.

Beyond MR and mediation analysis, future microbiota–ND studies should incorporate contemporary causal inference strategies. Triangulation of evidence can improve causal inference by integrating results from approaches with different and largely unrelated sources of bias, such as longitudinal human cohorts, MR, negative-control analyses, cross-cohort replication, FMT/HMA experiments, and metabolite perturbation studies. The directed acyclic graphs (DAGs) can make causal assumptions explicit and help distinguish confounders, mediators and reverse-causal pathways involving host genotype, diet, medication use, disease stage, gut microbiota, metabolites, immune activation, and ND phenotypes. The target trial emulation provides a useful framework for translating observational microbiome data into well-defined causal questions by specifying eligibility criteria, intervention or exposure strategies, assignment time, follow-up, outcomes, causal contrasts, and analysis plans before estimation. Finally, microbiome-specific causal discovery and constraint-based microbiome-metabolome modeling may help prioritize mechanistic hypotheses, but such computational outputs should be interpreted as hypothesis-generating and validated experimentally.

In summary, although the above theoretical statistical methods show certain advantages in causal inference, they are usually conducted for a single disease, resulting in low inference efficiency. Moreover, they struggle to integrate the large amount of accumulated multi-source heterogeneous association data involving host genes, microbes, and diseases. There is an urgent need for methodological innovation in the field.

## 3. Evolutionary Analysis of Causal Mechanisms

In recent years, numerous studies have revealed that the occurrence and development of human diseases are closely related to the process of human evolution. Many diseases are essentially the result of evolutionary trade-offs within the human genome, which has given rise to the emerging discipline of Evolutionary Biomedicine [26]. Since evolutionary information naturally carries causal temporal attributes, studying human diseases from an evolutionary perspective helps in deciphering disease mechanisms, clarifying genotype-phenotype causal associations, discovering innovative drugs, and achieving health security [26,27].

Human Accelerated Regions (HARs) are a class of genomic regions that exhibit high sequence conservation across mammals or even broader vertebrates but show significantly accelerated evolutionary characteristics in the human lineage. Research on HARs can assist in identifying human-specific genetic features and linking them to human-specific phenotypes. By comparing the genomes of humans and their close relatives, Pollard et al. identified 202 HARs in 2006 [28]. Subsequently, in 2016, Doan et al. integrated previous findings to construct a more comprehensive dataset containing 2737 HARs [29]. These studies found that although the vast majority of HARs are not in exonic regions, meaning they do not directly alter protein sequences [30], several HARs function as genetic regulators. For example, HAR1, defined by Pollard et al., encodes a long non-coding RNA (lncRNA) that functions in the formation of cerebral cortical patterns and layout [28]. Thus, HAR1 is best regarded as a representative example of a human-accelerated noncoding RNA with neurodevelopmental relevance, rather than direct evidence that HAR lncRNAs universally control gut microbial metabolism. Given that most HARs are noncoding, their influence on microbiota-associated metabolism is likely indirect: HAR-associated enhancers or lncRNAs may alter host transcriptional programs, such as those regulating epithelial barrier integrity, mucin and glycan secretion, or immune tone, which subsequently reshape the host niche and microbial composition [31,32]. Some HARs function similarly to enhancers, which can remotely regulate gene expression levels [33]. These HARs play important regulatory roles in human development, including central nervous system development [34]. Transgenic studies show that HARs can trigger human-like developmental processes in mouse embryos. Some HARs overlap with the positions of neurotransmitter receptor genes. Gene Ontology (GO) analysis shows that genes near HARs are enriched with neurodevelopment-related functions. This series of studies suggests that the genetic changes brought by HARs may have promoted the evolution of the human nervous system [34,35,36], endowing humans with unique cognitive abilities.

In fact, HARs are closely related to cognitive impairments and neurological diseases. In 2015, Xu et al. found that HARs are enriched with SNPs associated with schizophrenia [37]. In 2017, Srinivasan et al. observed the same phenomenon in an independent HAR dataset [38]. Additionally, studies have found that genes and mutations associated with autism are also located in HARs [29]. We analyzed the association between known disease genes and HARs and found that HARs are enriched with genes related to psychiatric/neurological diseases, such as AD and PD [36]. It has been shown that not only do 32.42% of marketed drugs target genes near HARs, but all second-generation antipsychotics target at least one HAR gene, highlighting the potential of HAR genes as drug targets [36]. In 2022, Erady et al. found that HARs are not only enriched with schizophrenia and bipolar disorder-related loci, but the transcripts of novel open reading frames (nORFs) and transposons contained within them may also be related to mental illness [39]. The potential close relationship between HARs and psychiatric diseases has inspired related drug target discovery. Erady et al.’s research found that the structures of proteins encoded by some HAR-nORFs can be predicted, making them potential targets for treating mental illness [39].

Our previous study showed that HARs are enriched with genetic factors that regulate gut microbiota, and some HAR-regulated gut microbes are associated with NDs (Table 1) [40]. As illustrated in Table 1, which is primarily derived from our previous study [40] and reorganized here with supporting literature, we summarize candidate HAR-microbiota–ND axes. It should be noted that this table does not present a new analysis performed for this Perspective. Rather, the listed associations integrate reported HAR-related host genetic factors, published host genome–microbiome associations, curated or literature-supported microbe-ND associations, and comparative evidence for rapid microbial change during human evolution where available [40,41]. For example, clinical studies show that the gene *TLR4*, located in HARs, can mediate inflammatory responses in both AD [42] and PD [43]. The abundance of *Bacteroides*, which is regulated by *TLR4*, is negatively correlated with the risk of AD and PD [41]. Furthermore, the HAR gene *FTO* is strongly associated with AD [44], and the abundances of the gut microbes *Helicobacter bilis* and *Lactobacillus reuteri*, which are regulated by *FTO*, are closely related to the occurrence of AD and PD [43]. Notably, the opposite abundance changes for these gut microbes were observed in chimpanzees [45]. Human-specific genes and gut microbiota form co-evolutionary units that mediate the development of NDs [40]. Nevertheless, the relationship between HARs and the microbiota remains uncertain and is highly speculative.

The associations summarized in Table 1 were extracted from our previous work and supporting literature, and reorganized here as illustrative candidate axes. Briefly, host genetic factors located within or near HARs were linked to gut microbial taxa based on published host genome–microbiome association studies; microbial taxa were then connected to NDs using curated microbe-disease resources and disease-specific clinical or experimental reports.

To effectively bridge the gap between evolutionary genomic anchors and dynamic metabolic phenotypes, the proposed framework requires a stepwise computational integration strategy [46,47,48,49,50,51,52]. First, HARs can be mapped to putative target genes using genomic proximity and tissue-specific regulatory annotations (e.g., eQTLs). Second, host genome–microbiome association studies and paired microbiome-metabolome data can link these genes to specific microbial taxa and downstream metabolites. Third, these multi-omics connections can be structured into knowledge graphs and evaluated using robust statistical tools, such as longitudinal mediation analysis, MR, or Bayesian networks. Finally, specific microbe–metabolite links are prioritized using mechanism-oriented metabolic modeling or representation learning. Importantly, these computational outputs should be utilized strictly to nominate candidate axes for downstream experimental testing, rather than to claim causality on their own.

## 4. Metabolite-Mediated Pathogenic Mechanisms of Gut Microbiota

Increasing evidence suggests that the gut microbiota does not influence the host primarily through its mere presence or absence, but rather through its metabolic outputs and related small-molecule effectors [53,54,55,56]. Metabolites can therefore be regarded as important molecular intermediates of host–microbiota interactions, translating ecological disequilibrium of the gut microbiota into biologically perceivable and transmissible pathological signals [55,56]. Current evidence indicates that lipopolysaccharide (LPS), short-chain fatty acids (SCFAs), and microbiota-modified bile acids are among the major mediators linking host genetic background, gut microbes, and disease phenotypes [53,54,55,56,57]. These metabolites can act in concert through the intestinal barrier, immune cells, the vagus nerve, the blood-brain barrier, and peripheral metabolic networks, thereby shaping the course of neurodegenerative pathology [3]. On this basis, gut microbe-associated pathogenesis can be summarized as a four-part relationship: host receptor/signaling pathway activation, microbial compositional change, metabolite-spectrum remodeling, and disease phenotype formation. Within this framework, metabolites are no longer viewed merely as secondary byproducts of microbial alteration, but rather as critical mediators that connect dysbiosis to disease progression in the host. Conversely, microbial metabolites such as SCFAs can feed back onto host chromatin and RNA regulatory programs through epigenetic mechanisms, including histone modification and chromatin accessibility, creating a potential bidirectional regulatory loop [31,32]. Therefore, we infer that metabolites can be utilized to elucidate the causal mechanisms by which the gut microbiotas mediate the association between evolutionary genetic factors and NDs.

LPS provides a representative example in this area. LPS is derived primarily from the outer membrane of Gram-negative bacteria, including *Proteobacteria* whose abundance is regulated by the HAR-located gene *TLR4* [40]. LPS represents a structurally diverse class of large, complex bacterial glycolipids/glycoconjugates, generally composed of lipid A, a core oligosaccharide, and O-antigen polysaccharide. When the intestinal barrier is compromised and gut permeability increases, luminal LPS is more likely to enter the portal and systemic circulation, where it is sensed by the LBP-CD14-MD-2/TLR4 receptor recognition complex, thereby initiating innate immune activation [58,59]. TLR4 then signals through both the MyD88-dependent and TRIF-dependent pathways to activate NF-κB and related inflammatory cascades, inducing the release of cytokines such as IL-1β, IL-6, and TNF-α, together with sustained microglial activation and amplification of neuroinflammation [58,59]. Experimental studies further show that systemic LPS exposure can provoke neuroinflammation and exacerbate Aβ and phosphorylated Tau (p-Tau) pathology [60,61]. In PD, increased LPS burden, intestinal leakage, and *TLR4*-mediated inflammation are thought to act together to promote abnormal α-synuclein aggregation in both the gut and the brain, as well as broader neurodegenerative changes; notably, in transgenic and toxin-induced models, modulation of the microbiota or inhibition of the LPS-TLR4 axis attenuates motor deficits and neuroinflammation [62,63,64]. Mechanistically, these findings support a model in which the HAR gene *TLR4* shapes microbial composition, whereas dysbiosis in turn sustains and amplifies *TLR4*-associated inflammatory signaling through LPS, ultimately translating intestinal immune and metabolic imbalance into neurodegenerative phenotypes such as AD or PD. In this sense, LPS can be viewed as a molecular bridge by which gut microbial dysbiosis is converted into neuropathological change. However, it should be noted that the TLR4-LPS axis is presented here primarily as an illustrative example rather than a universal mechanism. It demonstrates how a candidate genetic factor, a microbial group, a defined microbial product, and a host inflammatory pathway can be organized into a testable hypothesis. Other HAR–microbiota–metabolite axes may involve fundamentally different metabolites, receptors, tissues, or disease stages, and therefore must be evaluated independently.

From a drug discovery perspective, this host–microbe–metabolite causality provides a useful pathway for identifying novel interventions. Using the hypothetical TLR4-LPS axis as an example, once validated in specific patient subgroups, therapeutic strategies could target multiple downstream levels: reducing LPS-producing taxa via precision microbiota modulation, enhancing intestinal barrier integrity, or inhibiting host TLR4/NF-κB signaling. Rather than targeting HARs directly, this strategy leverages HAR-linked multi-omics data to uncover tractable downstream targets. It must be emphasized that these metabolite-based interventions are currently hypothetical and require extensive preclinical validation. Furthermore, such strategies must be highly context-specific, as the clinical impact of any given metabolite is likely modulated by the host’s genetic background, disease stage, and the prevailing microbial community structure.

It’s worth noting that microbial metabolites should not be viewed as the only route through which dysbiosis may influence neurodegeneration. Rather, they represent one mechanistically tractable layer within a multi-layered host–microbiota system that also includes epithelial barrier integrity, innate and adaptive immunity, vagal signaling, endocrine regulation, systemic metabolism, blood–brain barrier function, and glial activation. Moreover, the biological effects of a given metabolite may vary by diet, age, medication exposure, host genotype, geography, microbial strain background, disease stage, and sampling or analytical platform. Therefore, metabolite-centered hypotheses require context-specific validation rather than assuming a universal dysbiosis–metabolite–ND pathway. The proposed evolutionary-metabolic framework is hypothesis-generating and has several limitations. It would be weakened if candidate HAR-linked host genetic factors do not reproducibly associate with microbial features or metabolite profiles in independent and ancestrally diverse cohorts; if microbial changes do not temporally precede or predict metabolite remodeling and ND-related phenotypes; if targeted manipulation of the implicated microbe or metabolite fails to modify disease-relevant cellular or animal phenotypes; or if the apparent associations are fully explained by diet, age, medication, lifestyle, disease stage, or population structure. Future validation should combine longitudinal human cohorts with host genotyping, strain-resolved metagenomics, targeted and untargeted metabolomics, and standardized neurological phenotyping. Only axes supported across multiple complementary lines of evidence should be advanced as candidate causal mechanisms.

When this framework is applied to future research, researchers should identify a HAR-linked host factor, nominate associated microbial taxa, and connect them to measurable metabolites. Second, temporal relationships must be tested using longitudinal human cohorts. Finally, specific microbe-to-metabolite links should be validated via strain-resolved metagenomics, and their causality rigorously evaluated by perturbing the microbe, metabolite, or host receptor pathway in controlled experimental systems (e.g., gnotobiotic or HMA models) to determine whether ND-relevant phenotypes change [9,10,11,12,50,51,52,57]. To fully realize this approach at a population level, the field must address the context-dependent nature of microbiome data. Moving forward, integrating ancestrally diverse cohorts, strain-resolved shotgun metagenomics, paired longitudinal metabolomics, and harmonized metadata, which subjected to the rigorous causal adjustment designs mentioned above, will be essential to bridge the gap between evolutionary priors and broad clinical translation. A practical application of this framework proceeds systematically from prioritization to perturbation. First, researchers should identify a HAR-linked host factor, nominate associated microbial taxa, and connect them to measurable metabolites. Second, temporal relationships must be tested using longitudinal human cohorts. Finally, specific microbe-to-metabolite links should be validated via strain-resolved metagenomics, and their causality rigorously evaluated by perturbing the microbe, metabolite, or host receptor pathway in controlled experimental systems (e.g., gnotobiotic or HMA models) to determine whether ND-relevant phenotypes change [9,10,11,12,50,51,52]. To fully realize this approach at a population level, the field must address the context-dependent nature of microbiome data. Moving forward, integrating ancestrally diverse cohorts, strain-resolved shotgun metagenomics, paired longitudinal metabolomics, and harmonized metadata will be essential to bridge the gap between evolutionary priors and broad clinical translation.

## 5. Conclusions

Establishing causality in gut microbiota–ND research has been hindered by the field’s reliance on correlational data and by the methodological limits of experimental models (FMT, HMA rodents) and statistical frameworks (MR, mediation analysis). In this Perspective, we have argued that progress requires reframing the question through two complementary and temporally ordered lenses. Evolutionary genetics, anchored by HARs, provides a principled basis for prioritizing human-specific host–microbe co-evolutionary units relevant to neurodegeneration, while microbial metabolites serve as the mechanistically testable effectors that translate dysbiosis into disease phenotypes. The TLR4-LPS axis illustrates how a HAR-related host gene, its regulated microbial taxa, and a defined metabolite can be assembled into a structure causal chain as a candidate framework extending from the gut to AD- and PD-relevant neuropathology. Importantly, HARs are proposed here as host-side evolutionary priors rather than deterministic drivers of microbiota composition or neurodegenerative disease. The TLR4-LPS axis is presented here primarily as an illustrative example rather than a universal mechanism. It is not intended to apply to all HAR-associated genes, microbial taxa, or neurodegenerative diseases; rather, it demonstrates the kind of mechanistic structure and testable hypothesis that future candidate axes should aim to achieve. Specific HAR–microbiota–ND links remain candidate hypotheses that require independent population-level and experimental validation.

Classical longitudinal and repeated-measures designs remain indispensable for causal inference in microbiome research. Time-series sampling, repeated-measures ANOVA-like analyses, linear mixed-effects models, generalized linear mixed models, longitudinal mediation analysis, Granger causality, and dynamic Bayesian networks can help evaluate whether microbial or molecular changes temporally precede disease-related phenotypes. These approaches are particularly important because microbiome and metabolome data are dynamic, individualized, and often affected by irregular sampling, missing time points, and within-subject correlation. In this context, an evolutionary- and metabolite-centered framework will not replace existing causal inference tools, but it can supply the biological priors they currently lack, and in doing so move gut microbiota–ND research from correlation toward mechanism and, ultimately, toward tractable therapeutic targets. In light of these unique evolutionary and functional attributes, HARs were selected as a starting evolutionary anchor in our framework because they capture human-lineage acceleration on otherwise conserved genomic backgrounds, are frequently noncoding regulatory elements, and are repeatedly implicated in human neurodevelopmental and neuropsychiatric traits. Nevertheless, we acknowledge that HARs are not the only evolutionary features relevant to the gut-microbiota–ND axis. Future studies should compare HAR-based prioritization with other evolutionary markers, including loci under recent positive selection, human-specific duplications or copy number changes, accelerated promoters/enhancers, conserved noncoding elements, pseudogenization events, and metabolism/immune-related selected loci such as LCT, FUT2, ABO, and HLA. Thus, HARs should be viewed as one tractable evolutionary prior rather than an exclusive explanation of host–microbiota co-evolution.

## Figures and Tables

**Table 1:** HAR-regulated gut microbiotas and associated neurodegenerative diseases.

Genetic Factors Located in HARs	Microbe Name	Associated Neurodegenerative Diseases	Rapid Change During Human Evolution	Strength of Evidence
*AVP*	*Desulfovibrio* sp.	Parkinson’s Disease	N/A	Stronger
*FTO*	*Helicobacter bilis*	Alzheimer’s Disease	Yes	Stronger
	*Lactobacillus reuteri*	Parkinson’s Disease	Yes	Hypothesis
rs483905	*Comamonas*	Parkinson’s Disease	N/A	Hypothesis
rs4880904	*Rhizobiales*	Alzheimer’s Disease,	N/A	Stronger
		Parkinson’s Disease		
*SORL1*	*Dialister*	Alzheimer’s Disease	Yes	Stronger
*TLR4*	*Bacteroidetes*	Alzheimer’s Disease,	Yes	Stronger
		Amyotrophic Lateral Sclerosis,		
		Parkinson’s Disease		
	*Proteobacteria*	Alzheimer’s Disease,	N/A	Hypothesis
		Parkinson’s Disease		

## Data Availability

No new data were created or analyzed in this study.
